# The Association Between Time in Range %, Measured by Continuous Glucose Monitoring (CGM) and Physical Health Agility Status Indices Amongst Older People with T2D: A Cross-Sectional Study

**DOI:** 10.3390/jcm13237089

**Published:** 2024-11-23

**Authors:** Yamit Basson-Shleymovich, Tal Yahalom-Peri, Michal Azmon, Tali Cukierman-Yaffe

**Affiliations:** 1 Epidemiology Department, School of Public Health, Faculty of Health, Tel Aviv University, Tel Aviv 6997801, Israel; tal.yahalom@gmail.com (T.Y.-P.); tcukierm@gmail.com (T.C.-Y.); 2 Clalit Health Services, Blumental Physical Therapy Clinic, Bnei-Brak 5126722, Israel; 3 Sheba Medical Center, Division of Endocrinology, Ramat-Gan 5266202, Israel; 4 Faculty of Health Sciences, Ariel University, Ariel 40700, Israel; michalazmon014@gmail.com; 5 Herczeg Institute on Aging, Tel-Aviv University, Tel Aviv 6997801, Israel

**Keywords:** type 2 diabetes, glucose control, continuous glucose monitoring (CGM), sarcopenia, aerobic capacity, strength, balance, frailty, falls

## Abstract

**Background:** Individuals with diabetes face a higher risk of mobility disabilities, frailty, and sarcopenia (physical health agility). Studies have shown an association between HbA1C levels and physical health agility status. There is less information available about the relationship with time in range (%) (TIR). The aim of this study was to evaluate the cross-sectional relationship between time in range % (TIR %) and physical health agility status among older adults with type 2 diabetes (aerobic capacity, gait speed, strength, balance, and frailty). **Methods:** A cross-sectional study was conducted among individuals with diabetes over the age of 60. Participants were equipped with a blinded continuous glucose monitor (CGM) system (Medtronic iPro™2 and CareLink™, Medtronic, Northridge, CA, USA)) for 1 week and underwent an elaborate physical functional assessment (physical health agility) at the beginning and end of that week. The relationship between various physical health agility indices and both TIR (%) and HbA1c was evaluated using linear regression, with adjustments made for age and sex. **Results:** This analysis pertains to 139 individuals over the age of 60 with type 2 diabetes. After adjustment for age and sex, a 1% higher TIR (70–180 mg/dL) was associated with a 0.169 better score on aerobic capacity and endurance (*p*-value = 0.023), 0.119 better score on muscle strength of the upper limb (*p*-value = 0.039), 0.164 better score on dynamic balance (*p*-value = 0.039), and 0.165 better score on the evaluation of fall risk and balance (*p*-value = 0.037). **Conclusions:** In older individuals with diabetes, a higher percentage of time in range (TIR) is linked to better physical agility status indices. Future research is needed to determine whether glucose levels are simply a marker of disease severity or if there is a potential causal relationship.

## 1. Introduction

**Figure 1:**
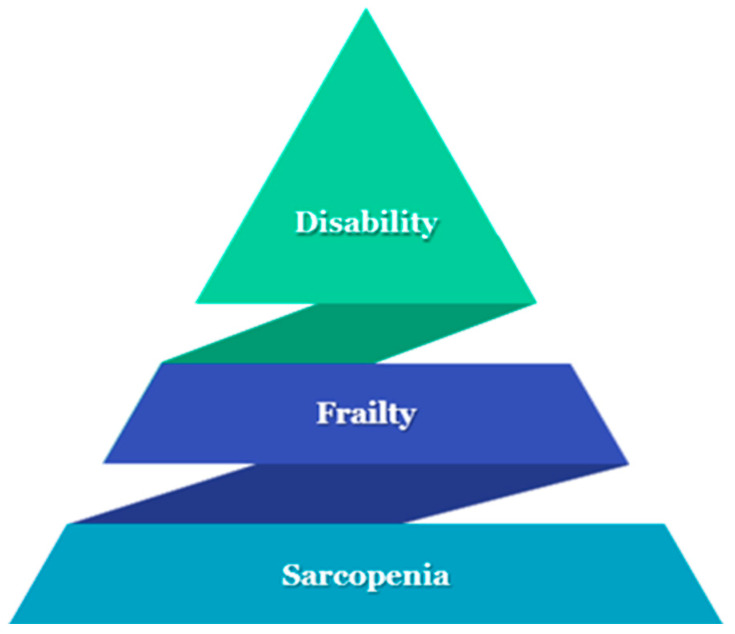
The iceberg of “physical health agility”: sarcopenia, frailty, and finally disability.

Diabetes is a significant public health challenge, linked to high rates of mortality, morbidity, hospitalization, and healthcare utilization [1]. The risk of diabetes is increased by age [2]. People with diabetes are at higher risk for impairment in physical health agility, mobility disability [3], frailty [4], and sarcopenia [5,6] (Figure 1).

“Sarcopenia” describes an age-related reduction in muscle mass and function [7]; it has received numerous definitions. One of the most widely used definitions for sarcopenia today is that of the European Working Group On Sarcopenia In Older People (EWGSOP2), which defines low muscle strength as the hallmark of sarcopenia, while muscle quantity or quality, as well as physical performance, are additional criteria for the confirmation of sarcopenia. It has been suggested to be both a cause and consequence of type 2 diabetes [8]. Reduced muscle mass and function can lead to decreased metabolic rate and physical activity, insufficient glucose removal, and potentially may lead to the development of diabetes [9]. There are many explanations for this relationship. A potential modifiable factor is glucose control. HbA1c reflects average glucose levels and is used to evaluate glycemic control and predict complications related to diabetes [10,11]. However, its accuracy in reflecting an individual’s glycemic control is limited, as it does not account for glycemic variability, acute fluctuations in glucose levels, or daily patterns of glycemia. These factors can contribute to acute events like hypoglycemia or hyperglycemia, which are associated with other comorbidities and complications of diabetes. Additionally, conditions that are relatively common in older adults, such as hemoglobinopathies, chronic kidney disease, and liver disease, can affect HbA1c measurements. Consequently, a patient’s glycemic status may range from excellent to poor, even among individuals with similar HbA1c levels [12,13]. The need for frequent and comprehensive glucose information with better hypo- or hyperglycemia detection, and a better understanding of an individual’s glycemic patterns, has led to the development of CGM technology. In recent years, several organizations have published consensus statements about the role of CGM and specific metrics to use for assessing overall glycemic status, hyperglycemia, hypoglycemia, and glycemic variability [1]. One of the important indices is a time in range % (TIR %) of 70–180 mg/dL (3.9–10 mmol/L). It has been popularized as an important metric to be derived from CGM data in order to better define glycemic control [14]. Studies have demonstrated a connection between time in range (TIR %) and diabetes complications [15,16,17,18].

There is limited information about the association between time in range (TIR %) and physical health agility indices in older adults with diabetes [6]. Thus, the purpose of this study is to evaluate the association between glucose levels, as measured by the time in range (TIR %), and physical abilities in individuals over the age of 60 with diabetes.

## 2. Materials and Methods

### 2.1. Participants

A cross-sectional study was conducted with 139 type 2 diabetics over the age of 60 between November 2019 and October 2021. Participants were recruited at the Sheba Medical Center during evaluation days and in diabetes clinics, as well as physiotherapy clinics and workshops that took place at community health service clinics, and through relevant forums and social media. Exclusions included individuals with significant hearing or visual impairments; those diagnosed with dementia or a cognitive impairment that, in the treating physician’s opinion, could affect their ability to sign a consent form; and those with major non-diabetes-related illnesses that were expected to reduce their life expectancy to within two years or interfere with study participation.

### 2.2. Study Design

**Figure 2:**
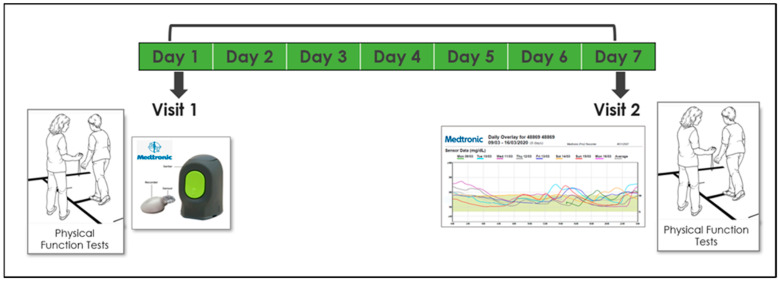
Timeline and description of the 7-day study design.

Participants were invited for 2 study visits, 1 week apart, at the center for successful aging with diabetes at the Sheba Medical Center in the city of Ramat-Gan. Measurements were conducted twice in order to increase reliability, reduce noise, and verify that the test–retest results were similar. On the first visit, subjects were connected to a CGM system (Medtronic iPro™2 and CareLink™, Medtronic, Northridge, CA, USA)which was installed on their left arm. They then underwent elaborate physical functional assessment by a physiotherapist, including an assessment of their aerobic capacity, gait speed, balance, muscle strength, physical activity, and frailty (see measurement section). Demographic and medical information (including diabetes-related variables) were also collected. In addition, subjects were asked to download a compatible application and document their glucose levels 3–4 times a day for calibration purposes as follows: in the morning before eating, one hour after a meal, and before sleep. On their second visit, a week after the first, participants underwent the same physical assessment and data from the iPro2 was downloaded. All data were collected, coded, and consolidated into a unified database (Figure 2).

### 2.3. Socio-Demographic Measures

Socio-demographic characteristics such as age, gender, education, marital status, employment status, ethnicity, and smoking status were gathered through a questionnaire. During the initial visit, anthropometric measurements, including self-reported weight and height, body mass index (BMI), and waist circumference, were recorded.

### 2.4. Glycemic Control (GC) Status

HbA1C was extracted from the patient’s medical reports. Glucose levels and Time in Range % (%TIR, between 70 and 180 mg/dL), were collected and downloaded from the CGM: Medtronic iPro™2 and CareLink™, Medtronic, Northridge, CA, USA.

### 2.5. Physical Capacity (PC) Assessment Battery

The physical and functional indices selected are relevant to key physical domains, are commonly utilized, and have strong validation. Below is a description of the indices used.

#### 2.5.1. Muscle Strength Assessment

1. The Hand Grip Strength Test was employed to evaluate upper body muscular strength. Maximum grip strength (in kilograms) was measured using the Jammer dynamometer manufactured by AliMed company, Dedham, Massachusetts, CA, USA. The test is performed with the hand in a neutral position and repeated three times. The final score represents the average strength in kilograms and is compared to normative values for the general population based on age and gender [19]. Excellent (r > 0.80) test–retest reproducibility [20] and excellent (r = 0.98) inter-rater reliability [21] have been reported for grip strength measured with the Jamar dynamometer. Longitudinal studies show that grip strength decreases after midlife, with the rate of decline accelerating as individuals age into their older years [22]. The grip strength assessment has demonstrated predictive validity, with lower values being associated with a higher risk of falls [23], disability, impaired health-related quality of life [24], and prolonged length of stay in hospital [25] as well as increased mortality [26].
2. The 30 s chair stand (STS) was used to assess lower limb muscle strength. The instructions were to get up from a sitting to standing position as many times as possible within 30 s without the use of hands or assistance. The score is given based on the number of times that the participant fails to reach full compliance. The strength of the lower limb muscles has a crucial impact on daily function; for example, when moving from a sitting position to a standing position, climbing up stairs, and walking [27].

#### 2.5.2. Aerobic Capacity Assessment

For aerobic capacity, the 6-min walk test (6 MWT) was administered [28]. The 6-min walk test (6 MWT) measures the distance an individual can walk over a 6-min period on a hard, flat surface, with the objective being to cover as much distance as possible. Participants are allowed to self-pace and rest as needed while walking back and forth along a marked pathway. In healthy adults, the typical 6-min walk distance ranges from 400 m to 700 m. Lower scores on the 6-min walk test are associated with a higher risk of falls, disability, frailty, hospitalization, and mortality [29,30].

#### 2.5.3. Gait Speed Evaluation

The 10-m walk test (10 MW) was conducted to determine gait speed [31]. The test evaluates the pace and number of steps required for a person to walk 10 m. A 10-m path is marked by two lines, with a chair positioned two meters beyond the end of the walkway. The subject begins the test two meters before the start of a 10-m runway and covers a total distance of 14 m (including two meters for acceleration at the start and two meters for deceleration at the end). The score is based on the time taken to walk the middle 10 m. The test is performed four times, with the first two attempts serving as practice; measurements are recorded during the third and fourth attempts. In addition to gait speed, the number of steps taken to cover the distance is also counted. Research indicates that better gait speed is associated with a reduced risk of functional decline, hospitalization, and mortality [32,33].

#### 2.5.4. Balance Assessment

Since the participants examined were at different levels of physical health agility, we utilized three different tests to assess their balance: the timed up and go (TUG) test, the Berg balance scale (BBS), and the four-square step test (FSST). Each contribute to a comprehensive evaluation.

The TUG test is quick, easy to perform, safe, functional, and efficient, measuring a range of mobility skills through timing. It provides information about the participant’s level of independence. The BBS, consisting of 14 tasks, evaluates both static and dynamic balance. Lower scores on this scale indicate impaired balance, which is associated with a higher risk of falls, hospitalizations, and mortality. The FSST assesses dynamic balance at a high functional level and is particularly useful for identifying individuals who struggle with changing directions.

The objective of the Timed up and Go TUG test (s) [29] is to evaluate a person’s ability to stand up, walk, turn around, and sit down safely and efficiently. It assesses a range of mobility skills. The participant is instructed to rise from a chair with their arms, walk 3 m, turn around, walk back, and then sit down again. Scoring is based on the time in seconds taken to complete the task, with scores categorized as follows:

1. Less than 14 s: Independent mobility.
2. 20–30 s: Dependent mobility; assistance needed for walking (50% use a cane, 40% use a walker, 10% need supervision). Many in this category may require help with transfers, using the toilet, and might not go outside the home alone.

Data indicate that the Timed Up and Go (TUG) test is both a reliable and valid measure of functional mobility and can be useful for tracking clinical changes over time [34,35,36].

The Berg Balance Scale (BBS) [37] consists of 14 tasks designed to assess both static and dynamic balance. Each task is scored from 0 to 4 points based on the quality and execution time, with a maximum possible score of 56 points. Scores are categorized as follows:

1. Scores below 36 indicate balance impairment and an increased risk of falls.
2. Scores between 37 and 45 suggest the need for a walking aid to ensure safe mobility.
3. Scores above 45 reflect independent walking ability without a heightened risk of falls.

The equipment used for the Berg Balance Test includes a step stool, a mat table, a chair with arms, a tape measure, a stopwatch, a pen, and a table. Studies have shown that individuals with lower scores, indicating impaired balance, are at a greater risk for falls, which can lead to hospitalizations and increased mortality.

Four Square Step Test (s) (FSST) assesses dynamic balance at a high functional level and involves walking forward, backward, left, and right over two 90 cm long and 2.5 cm high sticks that divide the floor into four squares. The participant starts in square 1, facing square 2. The goal is to walk as quickly as possible through the squares in the following sequence: from 1 to 2, then to 3, 4, back to 1, then to 4, 3, 2, and finally return to 1, all without touching the sticks. The score is based on the time taken to complete the entire route [38].

#### 2.5.5. Frailty Assessment

Frailty screening was conducted using the Fried Frailty Criteria [39], which include five components for assessing frailty:

1. Weight Loss: unintentional weight loss of more than 4.5 kg or 5% of body weight over the past year.
2. Poor Endurance and Energy: self-reported fatigue or low energy levels.
3. Low Physical Activity Level: assessed through a physical activity questionnaire.
4. Walk Time: taking more than 7 s to walk 3 m.
5. Low Grip Strength: measured relative to gender and body weight.

Pre-frailty is defined by the presence of two of these criteria, while frailty is identified by the presence of at least three components.

#### 2.5.6. Sample Size

The primary outcome was the correlation between TIR (%) and the score obtained on the 6-min walk (meters). The sample size of 139 enabled the detection of a correlation coefficient equal to or greater than 0.27 between the score obtained on the 6-min walk and the TIR %.

#### 2.5.7. Statistical Analysis

**Figure 3:**
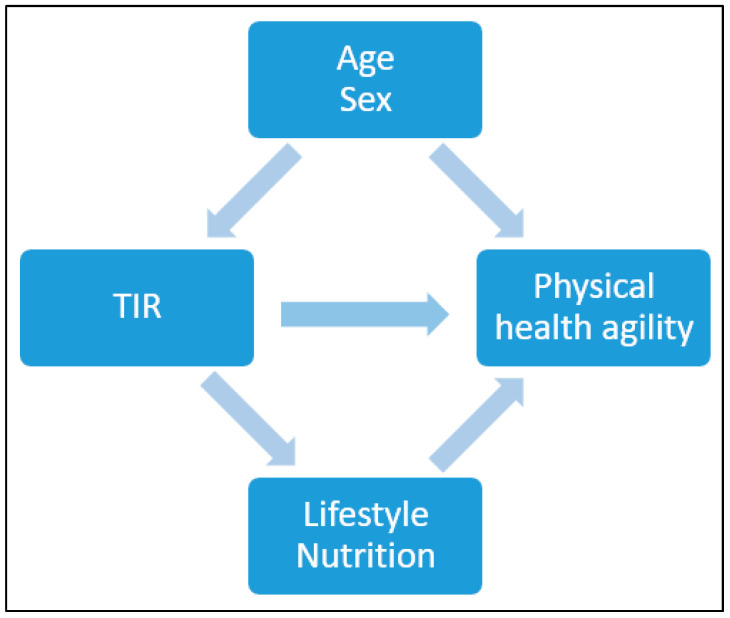
Possible confounders and mediator factors for TIR and physical health agility.

Continuous variables were summarized using means with standard deviations (SD), and binary variables were summarized using counts with percentages. Baseline variables were presented according to the TIR % and divided into 3 categories: >50, 50≥70, 70≤ (mg/dL). The difference in the distribution of the baseline variables was determined using a chi-square test for counts (percentages) and a one-way ANOVA test for means. To present the differences in the distribution of the baseline variables between all participants and those whom this analysis pertains to, we used *t*-test. The association between several physical indices and TIR (%)\HbA1C was assessed using Spearman correlation and linear regression, after adjusting for age and sex. Due to the limited sample size, adjustments were conducted for possible confounders and not for possible mediators (Figure 3). Additionally, in order to account for multiple comparisons a Bonferroni correction was also conducted.

## 3. Results

### 3.1. Dataset Description

This analysis pertains to the 144 individuals with diabetes that were eligible for the study. For technical reasons, five of them did not have a CGM measurement (the sensor fell of before the end of the week; the participant did not measure glucose manually; the sensor was not installed properly). Therefore, this analysis pertains to 139 consecutive participants over the age of 60 with type 2 diabetes, who conducted two physical capacity assessments and had CGM data available.

Table 1 depicts the baseline variables according to TIR % categories. The mean age was 71.29 years and 64.7% were male. There was a mean of 17.09 diabetes years for the entire cohort. The mean score of HbA1C was 7.06%. As can be seen, those with a TIR % above or equal to 70% had a shorter diabetes duration, lower HbA1C, were less likely to be insulin users, and had a lower risk for severe hypoglycemia.

Table 2 depicts physical and functional test scores according to TIR (%) categories. As can be seen, participants with a TIR % above or equal to 70% vs. those with a TIR < 70% had a better score on the TUG test (s) (a test that assesses the risk of falls and independent walking), the 360 turn test (s) (assessing dynamic balance), and were less likely to be frail.

### 3.2. The Association Between TIR (70–180 Mg\dL)/HbA1C and Several Physical Indices

**Figure 4:**
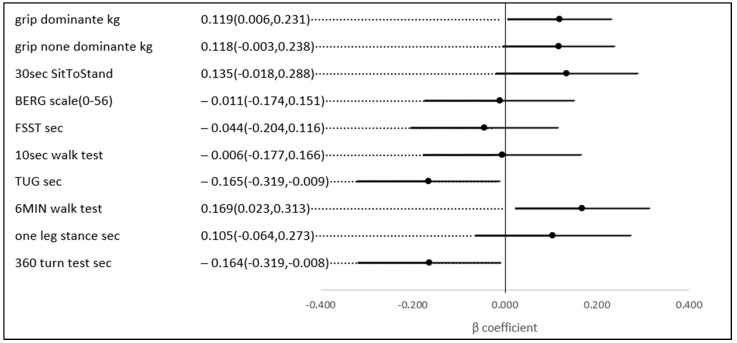
The association between a 1% higher TIR (70–180 mg\dL) and several physical indices, adjusted for age and gender.

No statistically significant univariate associations were found between HbA1c, TIR (%), and the scores of the physical and functional indices. However, in a multivariate analysis, after adjusting for age and gender (possible confounders, see Figure 3), a 1% higher TIR (70–180) (mg/dL) was associated with a 0.169 higher score on the 6-min walk score (meter) (*p*-value = 0.023), a test of aerobic capacity; a 0.119 higher score on the grip test (Kg) (*p*-value = 0.039), the hallmark of sarcopenia and a test of upper extremity strength; a 0.164 lower score on the 360-turn test (s) (*p*-value = 0.039); and 0.165 lower score on the TUG (s) (*p*-value = 0.037). The last two both assess dynamic balance and their score is a predictor of the risk of falls (Figure 4).

**Figure 5:**
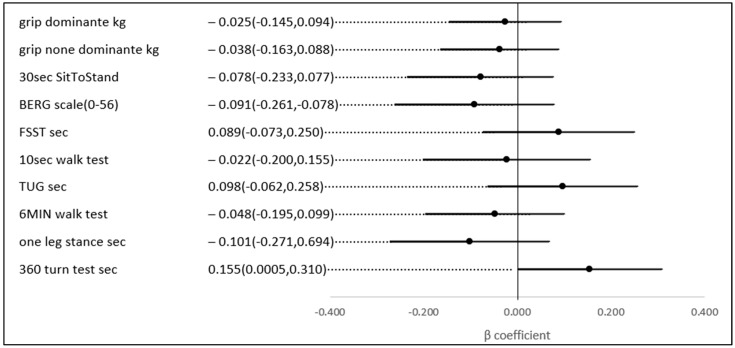
The association between a 1% higher HbA1C and several physical indices, adjusted for age and gender.

The same results were not observed for the relationship between HbA1C and most physical capacity tests; thus, the only statistically significant association noted was between HbA1C and the 360-turn test (s) (*p*-value = 0.049) (Figure 5).

## 4. Discussion

This analysis of 139 individuals over the age of 60 with type 2 diabetes reveals that a higher percentage of the time in range (TIR) is linked to improved scores on various indices of aerobic capacity, balance, and strength. After adjusting for age and gender, each 1% increase in TIR was associated with a 0.169 higher score on the 6-min walk test (a measure of aerobic capacity), a 0.119 higher score on the hand grip test (a key indicator of upper extremity strength and sarcopenia), a 0.164 improvement in the 360-turn test (assessing dynamic balance), and a 0.165 better score on tests evaluating fall risk and independent walking (TUG test, s).

Previous studies report similar results. The Women’s health and aging study II found that amongst 329 women aged from 70 to 79, a higher HbA1C category at baseline was associated with a higher incidence of walking difficulty and lower physical performance [40]. In older Korean men aged 65 years and older with type 2 diabetes, an HbA1c level above 8.5% was linked to a decline in lower limb muscle quality and physical performance measures [41]. The Baltimore Longitudinal Study of Ageing reported in Caucasian and Black individuals that knee extensor strength decreased progressively with increasing quartiles of HbA1c [42]. A study from China, conducted in 280 subjects over the age of 60 with type 2 diabetes, reported that individuals with higher TIR % were less likely to suffer from sarcopenia [6].

There are several possible explanations for the association between TIR % and physical health agility status. First, as this is a cross-sectional study it may well be that glucose control is a marker of a better self-care capacity; thus, people that have better lifestyle behaviors have better glucose control, engage in more physical activity, and therefore have better physical health agility indices. It may also be that people with lower physical health agility that are frail or pre-frail, engage in less physical activity, with a subsequent rise in glucose levels. Second, lower physical health agility indices in those with higher glucose levels may reflect an already recognized relationship with diabetes complications like retinopathy, nephropathy, and neuropathy. Third, it may be that glucose control may play a role in the development of sarcopenia. This may be mediated through several pathways: (1) nutritional deficiencies and the risk of malnutrition in people with type 2 diabetes [43,44]; (2) microvascular complications like retinopathy, nephropathy, and neuropathy [45,46]; (3) atherosclerosis and advanced glycation end products (AGE) [43,47], chronic, low-grade systemic inflammation [48,49,50], and poor glucose control and sensitivity to insulin.

## 5. Conclusions

This analysis of older people with type 2 diabetes over the age of 60, found a significant association between TR (%) and indices of aerobic, strength, and balance capacity. he fact that the results differed for TIR % and HbA1C may suggest that glycemic variability, or daily patterns of glycemia, which can be evaluated with CGM, are important in the physical health agility status of older people with diabetes. Prospective studies are needed to further understand this relationship.

### Study Limitations

This study has several limitations. First, the cross-sectional design limits its ability to establish temporality; thus, the direction of the relationship cannot be determined. Second, the use of a convenience sample of individuals who were either referred by a healthcare professional or self-referred, and the exclusion of individuals with significant cognitive and physical impairment, limits the generalizability of the results. We reported the associations between glucose indices and several physical indices, which may be a first step in better understanding the association between glucose levels and sarcopenia, frailty, and disability in older people with type 2 diabetes. Thus, given the limited sample size, we chose not to adjust for possible mediators like lifestyle and nutrition. Third, there was an under-representation of those with a relatively low TIR %, as well as those with low physical health agility indices. Finally, no comparison was conducted with a normoglycemic population thus limiting our ability to determine the differences between people with and without diabetes; however, our study provides insights with respect to the variability in older people with diabetes.

## Figures and Tables

**Table 1:** Baseline variables according to TIR (%) tertiaries.

	Total Mean	Total SD	>50 N = 9	50≤70 N = 27	70≥ N = 103	*p*
Male	90 (64.7)		7 (77.8)	18 (66.7)	61 (59.2)	0.464
Age	71.29	7.35	76.44 ± 7.75	69.63 ± 7.25	71.23 ± 7.10	0.067
Education (years)	15.48	3.46	14.1 ± 2.80	15.15 ± 3.24	15.63 ± 3.59	0.447
Dominant hand (R)	129 (92.8)		7 (77.8)	26 (96.3)	91 (88.3)	0.637
Weight (Kg)	80.57	14.53	71.66 ± 7.38	78.11 ± 11.44	81.52 ± 15.54	0.107
Hight (cm)	167.07	8.71	167.22 ± 8.65	169.37 ± 9.00	169.06 ± 8.75	0.810
Waist circumduction (cm)	106.67	14.12	98.91 ± 7.85	104.58 ± 10.26	106.32 ± 11.22	0.244
BMI	28.13	4.28	25.75 ± 3.46	27.25 ± 3.59	28.44 ± 4.40	0.108
HTN	90 (64.7)		5 (55.6)	22 (81.5)	65 (63.1)	0.156
Dyslipidemia	111 (79.8)		8 (88.9)	18 (66.7)	80 (77.7)	0.320
Diabetes duration (years)	17.09	10.32	20.33 ± 14.13	22.55 ± 9.45	15.60 ± 9.94	0.012
HbA1C	7.06	1.10	9.05 ± 1.81	7.77 ± 0.81	6.70 ± 0.79	0.000
Diabetes complication	134 (96.4)		9 (100)	23 (85.2)	96 (93.2)	0.257
Severe hypo	23 (16.5)		3 (33.3)	8 (29.6)	12 (11.7)	0.030
Insulin	41 (29.4)		5 (55.5)	19 (70.3)	17 (16.5)	0.000
Oral diabetes medications	118 (84.8)		5 (55.6)	23 (85.2)	85 (82.5)	0.117
Smoking	9 (6.4)		0 (0)	2 (7.4)	7 (6.8)	0.701
Former smoker	56 (40.2)		3 (33.3)	12 (44.4)	40 (38.8)	0.787
History of falls	28 (20.1)		2 (22.2)	4 (14.8)	21 (20.4)	0.813

Xx ± yy denotes mean ± SD; X(y) denotes N(%).

**Table 2:** Distribution of physical and functional scores according to TIR (%) tertiaries.

	Total Mean	Total SD	>50 N = 9	50≥70 N = 27	*p*
GRIP strength dominant hand (KG)	25.27	9.51	24.44 ± 10.77	25.63 ± 9.19	0.803
BERG total score	52.87	9.00	53.30 ± 5.99	52.84 ± 10.10	0.791
FSST time (s)	10.40	3.30	10.53 ± 3.97	10.28 ± 3.02	0.738
6 MWT (meter)	509.33	117.22	498.80 ± 135.07	518.73 ± 111.48	0.149
30 s sit to stand	16.32	5.77	15.21 ± 4.34	16.80 ± 6.09	0.235
TUG (s)	8.91	2.93	9.70 ± 4.17	8.56 ± 2.30	0.043
10 MWT (s)	8.01	2.18	8.24 ± 2.36	7.79 ± 1.80	0.208
One-leg stance (s)	17.61	10.43	15.22 ± 10.65	18.55 ± 9.99	0.236
360 turn test (s)	5.97	2.00	6.11 ± 1.91	5.78 ± 1.76	0.031
Physical activity questionnaire Total score	5.43	1.75	5.66 ± 1.85	5.49 ± 1.76	0.282
Prefrail	14 (10)		3 (11.11)	9 (8.1)	0.588
Frail	6 (4.3)		2 (7.4)	1 (1)	0.002
GRIP strength dominant hand (KG)	25.27	9.51	24.44 ± 10.77	25.63 ± 9.19	0.803
BERG total score	52.87	9.00	53.30 ± 5.99	52.84 ± 10.10	0.791
FSST time (s)	10.40	3.30	10.53 ± 3.97	10.28 ± 3.02	0.738
6MWT (meter)	509.33	0.4 ± 0.6 *	498.80 ± 135.07	518.73 ± 111.48	0.149

* Xx ± yy denotes mean ± SD; X(y) denotes N(%).

## Data Availability

The data that support the findings of this study are available on request from the corresponding author Tali Cukierman-Yaffe.

## References

[1] Zitkus B.S. (2014). Update on the american diabetes association standards of medical care. Nurse Pract..

[2] Kirkman M.S., Briscoe V.J., Clark N., Florez H., Haas L.B., Halter J.B., Huang E.S., Korytkowski M.T., Munshi M.N., Odegard P.S. (2012). Diabetes in older adults. Diabetes Care.

[3] Wong E., Backholer K., Gearon E., Harding J., Freak-Poli R., Stevenson C., Peeters A. (2013). Diabetes and risk of physical disability in adults: A systematic review and meta-analysis. Lancet Diabetes Endocrinol..

[4] Park S.W., Goodpaster B.H., Strotmeyer E.S., Kuller L.H., Broudeau R., Kammerer C., Huang E.S., Korytkowski M.T., Munshi M.N., Odegard P.S. (2007). Accelerated loss of skeletal muscle strength in older adults with type 2 diabetes: The health, aging, and body composition study. Diabetes Care.

[5] St-Onge M.-P., Gallagher D. (2010). Body composition changes with aging: The cause or the result of alterations in metabolic rate and macronutrient oxidation?. Nutrition.

[6] Ma G.C., Zou L.L., Dai W., Wang Z.Z., Cao Y.H. (2023). The association between glucose fluctuation with sarcopenia in elderly patients with type 2 diabetes mellitus. Eur. Rev. Med. Pharmacol. Sci..

[7] Cruz-Jentoft A.J., Baeyens J.P., Bauer J.M., Boirie Y., Cederholm T., Landi F., Martin F.C., Michel J.-P., Rolland Y., Schneider S.M. (2010). Sarcopenia: European consensus on definition and diagnosis: Report of the European Working Group on Sarcopenia in Older People. Age Ageing.

[8] Scott D., de Courten B., Ebeling P.R. (2016). Sarcopenia: A potential cause and consequence of type 2 diabetes in Australia’s ageing population?. Med. J. Aust..

[9] Mesinovic J., Zengin A., De Courten B., Ebeling P.R., Scott D. (2019). Sarcopenia and type 2 diabetes mellitus: A bidirectional relationship. Diabetes Metab. Syndr. Obes. Targets Ther..

[10] Nathan D.M., Genuth S., Lachin J., Cleary P., Crofford O., Davis M., Rand L., Siebert C. (1993). The effect of intensive treatment of diabetes on the development and progression of long-term complications in insulin-dependent diabetes mellitus. N. Engl. J. Med..

[11] UK Prospective Diabetes Study (UKPDS) Group (1998). Intensive blood-glucose control with sulphonylureas or insulin compared with conventional treatment and risk of complications in patients with type 2 diabetes (UKPDS 33). Lancet.

[12] Beck R.W., Connor C.G., Mullen D.M., Wesley D.M., Bergenstal R.M. (2017). The fallacy of average: How using HbA1c alone to assess glycemic control can be misleading. Diabetes Care.

[13] Suh S., Kim J.H. (2015). Glycemic variability: How do we measure it and why is it important?. Diabetes Metab. J..

[14] Danne T., Nimri R., Battelino T., Bergenstal R.M., Close K.L., DeVries J.H., Garg S., Heinemann L., Hirsch I., Amiel S.A. (2017). International consensus on use of continuous glucose monitoring. Diabetes Care.

[15] Hirsch I.B., Sherr J.L., Hood K.K. (2019). Connecting the dots: Validation of time in range metrics with microvascular outcomes. Diabetes Care.

[16] Lu J., Ma X., Zhou J., Zhang L., Mo Y., Ying L., Lu W., Zhu W., Bao Y., Vigersky R.A. (2018). Association of time in range, as assessed by continuous glucose monitoring, with diabetic retinopathy in type 2 diabetes. Diabetes Care.

[17] Krinsley J.S., Preiser J.-C. (2015). Time in blood glucose range 70 to 140 mg/dl >80% is strongly associated with increased survival in non-diabetic critically ill adults. Crit. Care.

[18] Omar A.S., Salama A., Allam M., Elgohary Y., Mohammed S., Tuli A.K., Singh R. (2015). Association of time in blood glucose range with outcomes following cardiac surgery. BMC Anesthesiol..

[19] Roberts H.C., Denison H.J., Martin H.J., Patel H.P., Syddall H., Cooper C., Sayer A.A. (2011). A review of the measurement of grip strength in clinical and epidemiological studies: Towards a standardised approach. Age Ageing.

[20] Mathiowetz V., Weber K., Volland G., Kashman N. (1984). Reliability and validity of grip and pinch strength evaluations. J. Hand Surg. Am..

[21] Peolsson A., Hedlund R., Oberg B. (2001). Intra- and inter-tester reliability and reference values for hand strength. J. Rehabil. Med..

[22] Bohannon R.W. (2008). Hand-grip dynamometry predicts future outcomes in aging adults. J. Geriatr. Phys. Ther..

[23] Sayer A.A., Syddall H.E., Martin H.J., Dennison E.M., Anderson F.H., Cooper C. (2006). Falls, sarcopenia, and growth in early life: Findings from the hertfordshire cohort study. Am. J. Epidemiol..

[24] Syddall H.E., Martin H.J., Harwood R.H., Cooper C., Sayer A.A. (2009). The SF-36: A simple, effective measure of mobility-disability for epidemiological studies. J. Nutr. Health Aging.

[25] Gale C.R., Martyn C.N., Cooper C., Sayer A.A. (2007). Grip strength, body composition, and mortality. Int. J. Epidemiol..

[26] Cooper R., Kuh D., Hardy R., Mortality Review Group, on behalf of the FALCon and HALCyon Study Teams (2010). Objectively measured physical capability levels and mortality: Systematic review and meta-analysis. BMJ.

[27] Jones C.J., Rikli R.E., Beam W.C. (1999). A 30-s chair-stand test as a measure of lower body strength in community-residing older adults. Res. Q. Exerc. Sport..

[28] Hamilton D.M., Haennel R.G. (2000). Validity and reliability of the 6-minute walk test in a cardiac rehabilitation population. J. Cardiopulm. Rehabil..

[29] Donovan K., E Lord S., McNaughton H.K., Weatherall M. (2008). Mobility beyond the clinic: The effect of environment on gait and its measurement in community-ambulant stroke survivors. Clin. Rehabil..

[30] Wolfson L., Whipple R., Amerman P., Tobin J.N. (1990). Gait assessment in the elderly: A gait abnormality rating scale and its relation to falls. J. Gerontol..

[31] Rossier P., Wade D.T. (2001). Validity and reliability comparison of 4 mobility measures in patients presenting with neurologic impairment. Arch. Phys. Med. Rehabil..

[32] Studenski S., Perera S., Patel K., Rosano C., Faulkner K., Inzitari M., Brach J., Chandler J., Cawthon P., Connor E.B. (2011). Gait speed and survival in older adults. JAMA.

[33] Fritz S., Lusardi M. (2009). White paper: Walking speed: The sixth vital sign. J. Geriatr. Phys. Ther..

[34] Van Iersel M.B., Hoefsloot W., Munneke M., Bloem B.R., Rikkert M.G.M.O. (2004). Systematic review of quantitative clinical gait analysis in patients with dementia. Z. Fur Gerontol. Und Geriatr..

[35] Van Iersel M.B., Munneke M., Esselink R.A.J., Benraad C.E.M., Olde Rikkert M.G.M. (2008). Gait velocity and the Timed-Up-and-Go test were sensitive to changes in mobility in frail elderly patients. J. Clin. Epidemiol..

[36] Van Iersel M.B., Ribbers H., Munneke M., Borm G.F., Rikkert M.G.O. (2007). The effect of cognitive dual tasks on balance during walking in physically fit elderly people. Arch. Phys. Med. Rehabil..

[37] Berg K.O., Wood-Dauphinee S.L., Williams J.I., Maki B. (1992). Measuring balance in the elderly: Validation of an instrument. Can. J. Public. Health.

[38] Dite W., Temple V.A. (2002). A clinical test of stepping and change of direction to identify multiple falling older adults. Arch. Phys. Med. Rehabil..

[39] Fried L.P., Tangen C.M., Walston J., Newman A.B., Hirsch C., Gottdiener J., Seeman T., Tracy R., Kop W.J., Burke G. (2001). Frailty in older adults: Evidence for a phenotype. J. Gerontol. A Biol. Sci. Med. Sci..

[40] Kalyani R.R., Tian J., Xue Q., Walston J., Cappola A.R., Fried L.P., Brancati F.L., Blaum C.S. (2012). Hyperglycemia and incidence of frailty and lower extremity mobility limitations in older women. J. Am. Geriatr. Soc..

[41] Yoon J.W., Ha Y.-C., Kim K.M., Moon J.H., Choi S.H., Lim S., Park Y.J., Lim J.Y., Kim K.W., Park K.S. (2016). Hyperglycemia Is Associated with Impaired Muscle Quality in Older Men with Diabetes: The Korean Longitudinal Study on Health and Aging. Diabetes Metab. J..

[42] Kalyani R.R., Metter E.J., Egan J., Golden S.H., Ferrucci L. (2015). Hyperglycemia predicts persistently lower muscle strength with aging. Diabetes Care.

[43] Sinclair A.J., Rodriguez-Mañas L. (2016). Diabetes and frailty: Two converging conditions?. Can. J. Diabetes.

[44] Velázquez-Alva M.C., Irigoyen-Camacho M.E., Zepeda-Zepeda M.A., Lazarevich I., Arrieta-Cruz I., D’Hyver C. (2020). Sarcopenia, nutritional status and type 2 diabetes mellitus: A cross-sectional study in a group of Mexican women residing in a nursing home. Nutr. Diet..

[45] Fowler M.J. (2008). Microvascular and macrovascular complications of diabetes. Clin. Diabetes.

[46] Nomura T., Ishiguro T., Ohira M., Ikeda Y. (2017). Diabetic polyneuropathy is a risk factor for decline of lower extremity strength in patients with type 2 diabetes. J. Diabetes Investig..

[47] Nowotny K., Jung T., Höhn A., Weber D., Grune T., Nowotny K., Jung T., Höhn A., Weber D., Grune T. (2015). Advanced glycation end products and oxidative stress in type 2 diabetes mellitus. Biomolecules.

[48] De Rekeneire N., Peila R., Ding J., Colbert L.H., Visser M., Shorr R.I., Kritchevsky S.B., Kuller L.H., Strotmeyer E.S., Schwartz A.V. (2006). Diabetes, hyperglycemia, and inflammation in older individuals: The health, aging and body composition study. Diabetes Care.

[49] Vozarova B., Weyer C., Lindsay R.S., Pratley R.E., Bogardus C., Tataranni P.A. (2002). High white blood cell count is associated with a worsening of insulin sensitivity and predicts the development of type 2 diabetes. Diabetes.

[50] Haddad F., Zaldivar F., Cooper D.M., Adams G.R. (2005). IL-6-induced skeletal muscle atrophy. J. Appl. Physiol..

